# Characterization and Genomic Analysis of *Arthrobacter* sp. SF27: A Promising Dibutyl Phthalate-degrading Strain

**DOI:** 10.2174/0113892029343036250210044540

**Published:** 2025-03-14

**Authors:** Ekaterina Korsakova, Yulia Nechaeva, Elena Plotnikova, Olga Yastrebova

**Affiliations:** 1 Laboratory of Microbiology of Technogenic Ecosystems, Institute of Ecology and Genetics of Microorganism UB RAS, Perm, Russia

**Keywords:** Phthalic acid esters, dibutyl phthalate, degradation, metabolic pathways, *Arthrobacter*, genome, *pht* gene cluster

## Abstract

**Background:**

Phthalic acid esters (PAEs) are widely used chemical compounds in various industries. However, PAEs are also a major source of pollution in soil and aquatic ecosystems, posing a significant environmental threat. Microbial degradation is a very effective way to remove phthalic acid esters from a polluted environment.

**Objectives:**

The aims of this study were to investigate the ability of the strain *Arthrobacter* sp. SF27 (=VKM Ac-2063) to degrade PAEs (specifically, dibutyl phthalate (DBF)); to annotate the complete genome of the strain SF27 (GenBank accession number GCA_012952295); to identify genes (gene clusters) potentially involved in the degradation of DBF and its major degradation product, phthalic acid (PA).

**Methods:**

The ability of the strain SF27 to use DBP as the only source of carbon and energy was determined by cultivating it on a mineral medium containing 0.5–4 g/L DBP. The evaluation of the bacterial decomposition of DBP was carried out by GC-MS. The genome was annotated using the JGI Microbial Genome Annotation Pipeline (MGAP) (https://jgi.doe.gov/). Functional annotation was performed using various databases: KEGG, COG, NCBI, and GO. The Mauve program was used to compare the strain SF27 genome and the genomes of the closest DBP-degrading strains.

**Results:**

The strain *Arthrobacter* sp. SF27 is capable of growing on DBP as the sole source of carbon and energy at high concentrations (up to 4 g/L). The strain was able to degrade 60% of DBP (initial concentration of 1 g/L) and 20% of DBP (initial concentration of 3 g/L) within 72 hours. The genome analysis of the strain SF27 (GenBank accession number GCA_012952295) identified genes encoding hydrolases potentially involved in the initial stages of DBP degradation, leading to the formation of PA. Additionally, a cluster of *pht* genes encoding enzymes that are responsible for the transformation of PA into protocatechuic acid (PCA) has been identified and described in the genome. Based on genome analysis and cultural experiments, a complete pathway for the degradation of PA by the strain *Arthrobacter* sp. SF27 into basal metabolic compounds of the cell has been proposed.

**Conclusion:**

Based on the conducted research, it can be stated that the strain *Arthrobacter* sp. SF27 is an efficient *degrader* of *DBP*, promising for the development of biotechnologies aimed at the restoration of ecosystems contaminated with DBP.

## 1 INTRODUCTION

The challenge of recycling organic compounds that resist decomposition, exhibit toxic properties, and tend to accumulate in ecosystems is becoming increasingly urgent each year. Some of the most widely used synthetic chemicals in different industries are phthalic acid esters (PAEs), particularly dibutyl phthalate (DBP), diethyl phthalate (DEP), and dimethyl phthalate (DMF). These compounds are commonly used as plasticizers in the production of polyester fibers, polyvinyl chloride (PVC), polyethylene, and building materials [1]. Additionally, due to their high demand in modern large-scale production, phthalic acid esters are widespread soil and water ecosystem pollutants, posing a serious environmental problem [2, 3]. According to the Organization for Economic Co-operation and Development (OECD), global PAE consumption exceeds 5.5 million tons per year (https://www.oecd.org/en.html). Phthalate esters and their metabolites possess carcinogenic, hepatotoxic properties and can cause disruption in the human endocrine system [4, 5]. The United States Environmental Protection Agency (EPA) has included these compounds in its list of priority environmental pollutants (https://www.epa.gov/).

Moreover, due to the low rate of chemical hydrolysis and photolysis of PAEs under natural conditions, bacterial degradation is recognized as the most promising and environmentally friendly method of PAE utilization [6, 7]. The ability to degrade PAEs was found in bacteria isolated from wastewater, activated sludge, sea and river sediments, soils contaminated with plastic waste, and waste from the mining industry [8-11]. PAE-degrading bacteria of the genera *Bacillus* [12-14], *Pseudomonas* [10], *Paenarthrobacter* [15], *Acinetobacter* [16], *Gordonia* [17], including the *members of the genus Arthrobacter* [18-21] have been discovered and studied. Strains with tolerance and degradative activity to various PAEs with long, short and cyclic side chains have been characterized. Most PAE-degrading strains exhibit degradation efficiency at a level of up to 1 g/l PAE. A number of strains have higher tolerance and efficiency with respect to PAE, for example, the new PAE-degrading strain *Gordonia* sp. GZ-YC7 exhibited the highest di-(2-ethylhexyl) phthalate degradation efficiency under 1000 mg/L and the strongest tolerance to 4000 mg/L [22]. The study of the biodegradation potential of PAE-degrading bacteria is of great importance for evaluating their use for the bioremediation of PAEs-contaminated environments.

Bacterial degradation of phthalic acid esters involves two main stages: the transformation of PAEs into phthalic acid (PA) and further degradation of PA into basal metabolic compounds of the cell [23]. The metabolic pathway for PA degradation *via* 3,4-dihydroxyphthalate to PCA, followed by cleavage of the benzene ring of PCA through the *ortho*- or *meta*-pathway, has been described for bacteria of the genus *Arthrobacter* [18, 24]. There are a number of publications describing the genes (gene clusters) of *Arthrobacter* strains that control the transformation of DBP to PA and then, with subsequent degradation of PA through the formation of PCA and transformation of PCA into acetyl-CoA *via* the 3,4-dioxygenase pathway [18, 24, 25].

Previously, we isolated and characterized the strain *Arthrobacter* sp. SF27 which is capable of degrading various mono(poly)aromatic compounds, including polycyclic aromatic hydrocarbons (phenanthrene, naphthalene), as well as phthalic, protocatechuic, benzoic, and gentisic acids [26-28]. In the present study, the ability of the strain *Arthrobacter* sp. SF27 to utilize DBP as the sole carbon and energy source at high concentrations was demonstrated. The aim of this work was to study the genetic and degradation characteristics of the strain *Arthrobacter* sp. SF27, as well as to establish the metabolic pathway for DBP degradation.

## 2 MATERIALS AND METHODS

### 2.1 Bacterial Strain

The strain *Arthrobacter* sp. SF27 (=VKM Ac-2063) used in this work was previously isolated from soils polluted with waste from the chemical and salt mining and processing industries in the city of Berezniki (Perm Krai, Russia) [25]. According to the sequence similarity calculation from the EzBiocloud server, this strain was most closely related to *A. crystallopoietes* DSM 20117^T^ (99.85% similarity), which is part of the *A. globiformis* group [27].

### 2.2 Chemicals

Dibutyl phthalate (DBP), dimethyl phthalate (DMP), diethyl phthalate (DEP), and phthalic acid (PA) with a purity >98% were purchased from Sigma-Aldrich (USA) and used as substrates. All other chemical reagents were of analytical purity, and all solvents were of chromatographic grade.

### 2.3 Media and Cultivation Conditions

The strain was cultivated in a mineral Raymond medium (MRM), containing (g/L): NH_4_NO_3_ – 2.0, MgSO_4_ х 7H_2_O – 0.2, K_2_HPO_4_ – 2.0, Na_2_HPO_4_ – 3.0, CaCl_2_ х 6H_2_O – 0.01, Na_2_CO_3_ – 0.1 (pH 7.0) [29]. The MRM was supplemented with 2 ml of 1% MnSO_4_ х 2H_2_O and 2 ml of 1% FeSO_4_ х 7H_2_O. For solid media, agar (Sigma-Aldrich, USA) was added to the liquid MRM before autoclaving to a final concentration of 15 g/L. The cultivation of microorganisms was carried out in a thermostat at 28°C.

The cultivation of microorganisms in the liquid MRM was carried out on a thermostatically controlled shaker (100 rpm) at 28°С.

The substrate specificity of bacteria was analyzed by culturing them in liquid Raymond medium with dibutyl phthalate (DBP), dimethyl phthalate (DMP), diethyl phthalate (DEP), or phthalic acid (PA) as the only source of carbon and energy.

### 2.4 Parameters of Bacterial Growth

The growth of bacteria was studied during batch culture in the liquid MRM [29] containing PA and DBP as the only source of carbon and energy. Exponential cultures grown on MRM containing PA as a substrate were used as inoculum. Bacterial growth on PA (1 g/L) and different concentrations of DBP (0.5, 1, 2, 3, 4 g/L) were evaluated in the MRM. Bacterial cultures were grown in 250-mL Erlenmeyer flasks (volume of the medium, 100 mL) with aeration on a thermostatically controlled shaker (100 rpm) at 28°C [30]. The optical density (OD_600_) of the culture liquid was determined using a UV Visible BioSpec-mini spectrophotometer (Shimadzu, Japan) at a wavelength of 600 nm in a cuvette with an optical path length of 1 cm. The specific growth rate (μ, h^–1^) was calculated according to the standard equation:

μ = *(lnB_2_ – lnB_1_)/(t_2_ – t_1_)*,

where *В*_1_ and *В*_2_ were optical densities of the culture at time moments *t*_1_ and *t*_2_, respectively [31].

### 2.5 PA Degradation and PCA Determination

PA and protocatechuic acid (PCA) in the growth medium were determined by high-performance liquid chromatography (HPLC) using an LC-20AD Prominence chromatograph (Shimadzu, Japan) with a column (C-18 150 × 4.6 mm; Sigma-Aldrich, United States) and UV-detector SPD-20A (at 205 nm) in the acetonitrile–0.1% H_3_PO_4_ system (70: 30). An 80% acetonitrile solution was used as the mobile phase at a flow rate of 1.0 mL/min at 40°C. The identification was carried out by comparing the peak release times of the extracts to standard solutions of PA and PCA at concentrations of 50 and 100 mg/L; the retention time (*tR*) of PA and PCA was 5.1 and 4.8 min, respectively [30]. The quantitative content was calculated using the LC solution software package (Shimadzu, Japan). The PA degradation rates were calculated according to the equation below:

Degradation rate (%) = *(1-C/C_0_) × 100%*,

where *C* and *C_0_* represent the PA content in inoculated and non-inoculated medium, respectively.

### 2.6 DBP Degradation

Evaluation of the bacterial decomposition of DBP was carried out by GC-MS. The strain was grown in liquid mineral Raymond medium (MRM, pH 7.0) [29] containing 1 g/L *ortho*-phthalate, in 250-mL Erlenmeyer flasks (volume of the medium, 100 mL) for 72 h at 28°C on a rotary shaker (150 rpm). The cells were harvested by centrifugation (12000 g, 5 min) and washed with MRM. Twice-washed cells were resuspended in liquid MRM (OD_600_ = 1.5). One milliliter of the culture was placed in Wheaton sample vials (Sigma-Aldrich, Germany) sealed with a PTFE-lined stopper. DBP was added to final concentrations of 0.5, 1, 2, and 3 g/L, and the culture was incubated at 28°C for three days with aeration on a rotary shaker (150 rpm). DBP was then extracted with an equal volume of hexane for 120 minutes on a shaker at 100 rpm. Residual water was removed from the samples by introducing anhydrous sodium sulfate.The analysis was performed on an Agilent GC 7890A MS 5975C Inert XL EI/CI gas chromatograph/mass spectrometer (United States) with a quartz capillary column HP-5MS SN US 15189741-1 (30 × 0.25 mm). The carrier gas was helium (1 mL/min), the evaporator temperature was 230°C, and the sample volume was 0.2 µL. Chromatographic peaks were identified based on retention times and mass spectra using the NIST 98 electronic mass spectra library [11]. The amount of DBP was assessed by the peak area on the calibration chart. The DBP degradation rates were calculated according to the equation below:

Degradation rate (%) = *(1-C/C_0_) × 100%,*

where *C* and *C_0_* represent the DBP content in inoculated and non-inoculated medium, respectively.

### 2.7 Genome Sequencing and Annotation

Genomic DNA was extracted according to Wilson (Wilson 1995) [32]. The draft genome of strain SF27 (=VKM Ac-2063) was obtained at the Genoanalytica Company, Russia (https://www.genoanalytica.ru) on an Illumina Hiseq 1500 platform. The quality control of the raw sequencing reads was performed using FastQC v0.11.7 [33]. The adapters were trimmed, and low-quality sequences were removed using Trimmomatic v0.36 [34]. The assembly was carried out using the SPAdes version 3.12.0 [35]. The genome was annotated using the JGI Microbial Genome Annotation Pipeline (MGAP) (https://jgi.doe.gov/) [36]. The predicted gene sequences were obtained performed by sequence alignment with NCBI nr (nonredundant protein database), Clusters of Orthologous Genes (COG), Gene Ontology (GO), and Kyoto Encyclopedia of Genes and Genome (KEGG) databases to obtain functional annotation information. The Mauve program was used to compare the strain SF27 genome and the genomes of the closest DBP-degrading strains. It visualizes an alignment as a series of conserved segments called Locally Collinear Blocks (LCBs), which are similar to synteny blocks (https://darling lab.org/mauve/download.html).

### 2.8 Statistical Analysis

All the experiments were conducted in triplicate. The results of growth and degradation experiments of the strain *Arthrobacter* sp. SF27 was processed using MS Office Excel 2013.

## 3 RESULTS AND DISCUSSION

### 3.1 DBP Biodegradation Kinetics by *Arthrobacter* sp. SF27 and Growth in a Mineral Medium with DBP

**Fig. (1):**
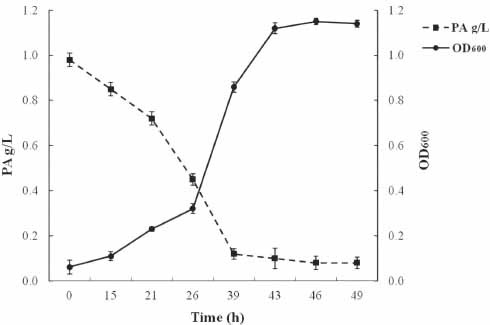
Growth curve and PA (1 g/L) degradation profiles of the strain *Arthrobacter* sp. SF27.

The strain *Arthrobacter* sp. SF27 was found to be capable of growth on phthalic acid as the sole source of carbon and energy. Using the HPLC method, it was shown that the strain *Arthrobacter* sp. SF27 was capable of degrading 91.8% phthalic acid (initial concentration 1 g/l) within 46 hours of cultivation (Table **1** and Fig. **1**). It was found that when grown on PA, protocatechuic acid (PCA) was present in the culture medium of the strain at concentrations of 0.16 and 0.11 g/L *after* 39 h and 43 h *of incubation*.

**Fig. (2):**
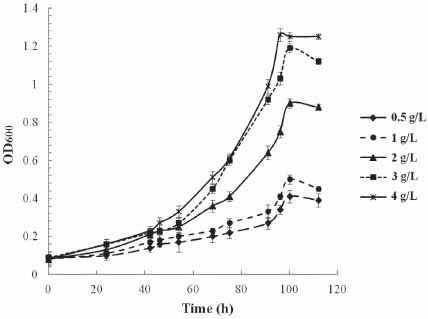
Growth curves of the strain *Arthrobacter* sp. SF27 at different DBP concentration.

The strain *Arthrobacter* sp. SF27 grew on phthalic acid esters ˗ DBP and DEP, but not on DMP. The most active growth of the strain was detected when cultivated on DBP as the only source of carbon and energy (Table **S1**). It was found that the strain SF27 was capable of growing on DBP at a concentration of 0.5 to 4 g/L (Fig. **1**). Increasing the concentration of DBP led to an increase in the maximum OD_600_ values of the culture from 0.41 to 1.26, as well as an increase in the specific growth rate of the strain from 0.024 to 0.032 h^-1^ (Table **1** and Fig. **1**). When the strain *Arthrobacter* sp. SF27 was grown on phthalic acid (1 g/L) as a substrate, and the specific growth rate was higher than on DBF at the same concentration, reaching 0.073 h^-1^ (Table **1** and Fig. **2**). The strain *Arthrobacter* sp. was described with maximum biomass accumulation observed after 120 hours at a DBP concentration of 0.6 g/L. There known strains *Arthrobacter* sp. ZJUTW and *Arthrobacter* sp. Z2 can grow on DBP at concentrations of 1 g/L and 0.5 g/L, in 18 and 48 h, respectively [18, 20]. To our knowledge, the growth of bacteria of the genus *Arthrobacter* on DBP at concentrations above 1 g/L has not been previously described.

The results of the experiment with washed cells showed that the strain SF27 was capable of partial degradation of DBP at a concentration of up to 3 g/L. The percentage of DBP degradation decreased with increasing DBP concentration from 60% at 0.5 g/L to 20.6% at 3 g/L DBP. The degradation rate also reduced from 57.6 to 14.5% within 72 h (Table **1**). The literature reports bacterial degradation of DBP at high concentrations. The strain *Bacillus* sp. degraded 2.783 g/L of DBP within 72 hours [12], *Pseudarthrobacter defluvii* strain E5 degraded 50% of DBP at a concentration of 1.2 g/L within two days [37], and *Pseudomonas* sp. V21b degraded DBP at a concentration of 2 g/L. Previously studied *Arthrobacter* strains are capable of partial or complete degradation of DBP at concentrations up to 1 g/L [38]. *Arthrobacter* sp. SF27 degraded 20.6% of DBP at a concentration of 3 g/L, which, to our knowledge, was demonstrated for the first time in bacteria of the genus *Arthrobacter*. Thus, it was shown that the strain SF27 actively grew at 3 and 4 g/L DBP, indicating extreme tolerance to DBP stress. It was also found that the strain SF27 degraded DBP most efficiently at lower concentrations (0.5 and 1 g/L DBP). In natural environments, DBP concentrations are most often present at low levels (<1 g/L). It indicated that the strain *Arthrobacter* sp. SF27 may be promising for DBP biodegradation.

### 3.2 Genome Analysis of *Arthrobacter* sp. SF27

**Fig. (3):**
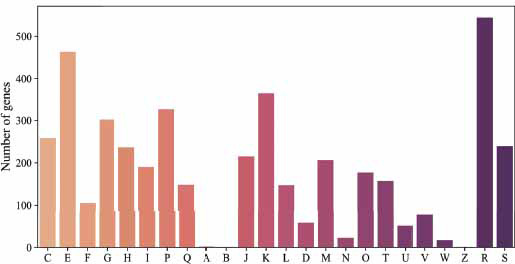
COG function classification of the strain *Arthrobacter* sp. SF27. **Cellular processes and signaling**: (D) Cell cycle control, cell division, chromosome partitioning. (M) Cell wall/membrane/envelope biogenesis. (N) Cell motility. (O) Post-translational modification, protein turnover, and chaperones. (T) Signal transduction mechanisms. (U) Intracellular trafficking, secretion, and vesicular transport. (V) Defense mechanisms. (W) Extracellular structures. (Y) Nuclear structure. (Z) Cytoskeleton. **Information storage and processing**: (A) RNA processing and modification. (B) Chromatin structure and dynamics. (J) Translation, ribosomal structure and biogenesis. (K) Transcription. (L) Replication, recombination and repair. **Metabolism**: (C) Energy production and conversion. (E) Amino acid transport and metabolism. (F) Nucleotide transport and metabolism. (G) Carbohydrate transport and metabolism. (H) Coenzyme transport and metabolism. (I) Lipid transport and metabolism. (P) Inorganic ion transport and metabolism. (Q) Secondary metabolites biosynthesis, transport, and catabolism. **Poorly characterized**: (R) General function prediction only. (S) Function unknown.

The complete genome sequence of the strain *Arthrobacter* sp. SF27 (=VKM Ac-2063) was deposited into DDBJ/EMBL/GenBank under the accession number GCA_012952295.1 The genome size of the strain SF27 was 4.96 Mbp (124 scaffolds). The DNA G+C content of this strain calculated from the genome sequence was 64.3%. The chromosome contains 4759 protein-encoding genes, 3 rRNA clusters (5S, 16S, and 23S) and 49 tRNAs. Among all the predicted CDSs (4759), 3580 genes (73.86%) were classified into 24 different categories of clusters of orthologous groups (COGs) (Fig. **3**). The most abundant COG categories were category R with general function prediction only (544), amino acid transport and metabolism (category E, 463 genes), transcription (category K, 365 genes), inorganic ion transport and metabolism (category P, 326 genes), carbohydrate transport and metabolism (category G, 302 genes), and energy production and conversion (category C, 258 genes) (Fig. **3**).

Similarly, the KEGG pathways with high proportion were mainly metabolic pathways such as biodegradation and xenobiotic metabolism, energy metabolism and lipid metabolism, as well as amino acid and carbohydrate metabolism. Therefore, 74.85% of protein-coding genes had predicted functions, of which 1257 genes were classified using the KEGG pathways database. It was noted that 86 genes relate to the degradation of various xenobiotics. This indicates that the strain *Arthrobacter* sp. SF27 has the potential ability to degrade and metabolize different organic pollutants, such as aromatic compounds (phenanthrene, naphthalene, benzoic acid, phthalic and protocatechuic acids) (Table **S2**, Table **S3**). Functional genes information from the strain SF27 is worthy of further analysis and mining.

### 3.3 Genes Presumably Involved in DBP Degradation

There are a number of studies on bacteria of the genus *Arthrobacter* that have examined their ability to degrade phthalate esters [24, 25, 39]. A Mauve-based comparison was performed between the genomes of the strain *Arthrobacter* sp. SF27 (GCA_012952295), DBP-degrading strains of the genus *Arthrobacter: Arthrobacter* sp. 68b (plasmid p2MP, NZ_KJ410765), *A. keyseri* 12B (plasmid pRE1, AF331043), and its closest relative *A. crystallopoietes* DSM 20117^T^ (GCA_002849715) (Fig. **S1**), which can weakly degrade DBP (data not shown).

As is known, possible metabolic pathways for the biodegradation of DBP were proposed and divided into three steps: 1) DBP are converted to PA by the action of a hydrolase; 2) PA is converted to PCA by several enzymes encoded by the *pht* gene cluster; 3) PCA is transformed into acetyl-CoA, and then enters the tricarboxylic acid (TCA) cycle as shown for a number of gram-positive strains, including strains of the genus *Arthrobacter* [24, 25]. However, a complete DBP metabolic pathway is still required for the discovery of new and improved strains [13, 18].

In the first DBP degradation step, DBP can be degraded to monoesters and further to PA *via* the carboxylic ester hydrolase family, mainly including some esterases, lipases, cutinase, and alpha/beta hydrolases [14]. Based on genome annotation, *Arthrobacter* sp. SF27 had 1 copy of the esterase-encoding gene, 1 copy of the carboxylesterase-encoding gene, and 22 copies of α/β-hydrolase-encoding genes (Table **S4**), which signifies that strain SF27 has great degradation potential. It is worth noting the high similarity (99,63- 82,76%) of the detected α/β-hydrolase-encoding genes with homologous genes of the strain *Arthrobacter* sp. VKM Ac-2550 (GCA_002849715), isolated from the contaminated soil of Perm Krai (Russia) [27]. The ester hydrolase genes of strain SF27 had slightly lower similarity (98.75-81.33%) with the same genes of strain *A. crystallopoietes* DSM 20117^T^.

**Fig. (4):**
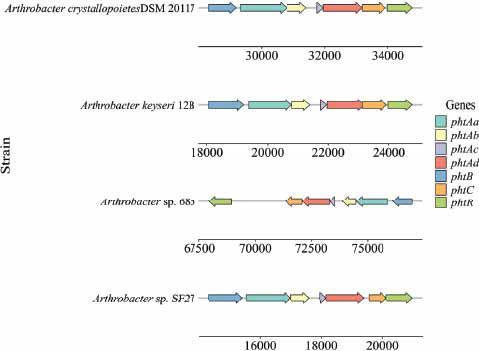
Cluster of genes involved in the degradation of phthalic acid in different strains of the genus *Arthrobacter*. *phtAa*, *phtAb*, *phtAc*, *phtAd* – genes encoding phthalate 3,4-dioxygenase; *phtB* – gene encoding phthalate-3,4-*cis*-dihydrodiol dehydrogenase; *phtС* – gene encoding 3,4-dihydroxyphthalate decarboxylase; *phtR* – transcription regulator of the IclR family.

Some gene clusters closely related to the biodegradation of DBP, including the *pht* and *pca* gene clusters, were identified in this study. We assume that the genome of the strain SF27 contains α/β-hydrolases encoding genes, which can be converted by DBP into PA, as previously described in various studies [18]. The *pht* gene cluster has been also found in the genome of *Arthrobacter* sp. SF27 (Table **S2**, Fig. **4**). The *pht* gene cluster has a size of 6660 bp and includes 7 *pht* genes encoding various subunits of 3 PA degradation enzymes, as well as a transcription regulator of the *IclR* family (Fig. **4**). The analysis revealed four *pthA* genes encoding the alpha- and beta-subunits of phthalate 3,4-dioxygenase, and the components ferredoxin and ferredoxin reductase. The search for homologous proteins showed the greatest similarity of the translated amino acid sequences of the strain *Arthrobacter* sp. SF27 with protein sequences of strains *A. keyseri* 12B, *Arthrobacter crystallopoietes* DSM 20117^T^ and *Pseudarthrobacter phenanthrenivorans* Sphe3. The level of similarity ranged from 89 to 100% (Table **S2**).

**Fig. (5):**
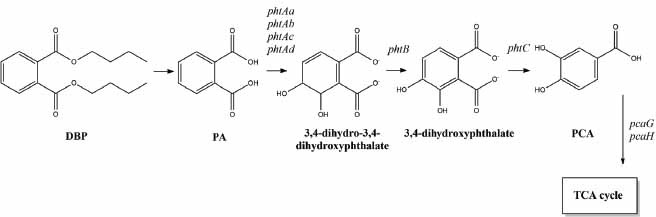
Pathway of PA degradation in the strain *Arthrobacter* sp. SF27. **Abbreviations:** DBP – dibutyl phthalate; PA– phthalic acid; PCA – protocatechuic acid; TCA cycle – tricarboxylic acid cycle.

In addition, the *phtB* gene encoding phthalate-3,4-*cis*-dihydrodiol dehydrogenase was discovered. The translated sequence of the *phtB* gene shares 97% similarity with the sequence encoding phthalate-*3,4-cis*-dihydrodiol dehydrogenase in *A. keyseri* strain 12B. The *phtC* gene, encoding 3,4-dihydroxyphthalate decarboxylase, exhibits 94% similarity to the translated gene sequence of strain *A. keyseri* 12B. The arrangement of *pht* genes in clusters of strains *Arthrobacter* sp. SF27, *A. keyseri* 12B, and *A. crystallopoietes* DSM 20117T is completely identical (Fig. **4**). Moreover, the structure of these clusters differs significantly from the clusters of *pht* genes in other genera of gram-positive bacteria capable of degrading PA [13, 17] Based on the data obtained, the degradation of PA to protocatechuate in the active DBP-degrading strain *Arthrobacter* sp. SF27 likely follows this pathway: PA is oxidized by phthalate 3,4-dioxygenase to 3,4-dihydro-3,4-dihydroxyphthalate, which is then dehydrogenated by phthalate-*3,4-cis*-dihydrodiol dehydrogenase to 3,4-dihydroxyphthalate, and subsequently decarboxylated by 3,4-dihydroxyphthalate decarboxylase to PCA (Fig. **5**). This pathway of PA degradation has been described for several gram-positive bacteria capable of degrading PA.

## CONCLUSION

This research demonstrated that the strain *Arthrobacter* sp. SF27 exhibited high degradation efficiency toward PAEs, specifically DBP and DEP. The strain was capable of growing on DBP as the sole source of carbon and energy at concentrations of up to 4 g/L, which has not previously been reported for the genus *Arthrobacter*. In addition, strain SF27 degraded DBP at concentrations ranging from 0.5 to 3 g/L within 72 hours, with higher efficiency at 0.5 and 1 g/L DBP. This is an important biodegradation characteristic of the strain, as DBP concentrations in natural environments are typically low (<1 g/L). The genome analysis of the strain *Arthrobacter* sp. SF27 (GenBank number GCA_ 012952295) identified 22 copies of α/β-hydrolase-encoding genes, the closest to similar genes of strains of the genus *Arthrobacter* (Table **S4**). DBP is known to be degraded into monoesters and further into PA by the carboxylic ester hydrolase family, which primarily includes esterases, lipases, cutinases, and alpha/beta hydrolase [14]. Presumably, the discovered hydrolases of the strain SF27 are involved in the first stages of DBP degradation, leading to the formation of PA. Further studies are needed to determine which hydrolase(s) are involved in phthalate ester degradation. A cluster of *pht* genes encoding enzymes that convert PA to PCA has been identified and described in the genome. Based on genome analysis and culture experiments, a complete pathway for PA decomposition by *Arthrobacter* sp. SF27 into basal metabolic compounds of the cell has been proposed. These findings suggest that *Arthrobacter* sp. SF27 is a highly active DBP-degrading strain with potential applications in biotechnologies for restoring ecosystems contaminated with DBP.

## STUDY LIMITATIONS

In the future, transcriptomic studies are needed to obtain more complete data on the metabolic pathway of DBP degradation in the *Arthrobacter* sp. SF27.

## Figures and Tables

**Table 1:** Kinetic parameters growth and degradation of phthalic acid (PA), dibutyl phthalate (DBP) by the strain *Arthrobacter* sp. SF27.

**Kinetic Parameters**	**PA** **(1 g/L)**	**DBP (g/L)**				
		**0.5**	**1**	**2**	**3**	**4**
Specific growth rate (h^-1^)	0.073 ± 0.007	0.024 ± 0.003	0.024 ± 0.002	0.029 ± 0.004	0.032 ± 0.006	0.032 ± 0.003
The maximum value of OD_600_	1.15	0.41	0.50	0.90	1.19	1.26
Degradation (%)*	91.8	60.0	47.4	44.0	20.6	nd-
Degradation rate (%)*	91,5 ±2,23	57,6 ±1,56	43,1 ±1,38	38,1 ±1,95	14,5 ±1,41	nd

Note: * – degradation was determined by 72 h of cultivation.

Designations: nd – not determined.

## Data Availability

The data supporting the findings of the article have been deposited in the GenBank nucleotide sequence database [https://www.ncbi.nlm.nih.gov/datasets/genome/] with accession numbers GCA_012952295.1.

## LIST OF ABBREVIATIONS

COG: Clusters of Orthologous Genes
DBP: Dibutyl Phthalate
DEP: Diethyl Phthalate
DMF: Dimethyl Phthalate
EPA: Environmental Protection Agency
GO: Gene Ontology
HPLC: High-performance Liquid Chromatography
KEGG: Kyoto Encyclopedia of Genes and Genome
LCBs: Locally Collinear Blocks
MGAP: Microbial Genome Annotation Pipeline
OECD: Organization for Economic Co-operation and Development
PAEs: Phthalic Acid Esters
PCA: Protocatechuic Acid
PVC: Polyvinyl Chloride
TCA: Tricarboxylic Acid

## References

[1] Naveen K.V., Saravanakumar K., Zhang X., Sathiyaseelan A., Wang M.H. (2022). Impact of environmental phthalate on human health and their bioremediation strategies using fungal cell factory- A review.. Environ. Res..

[2] Liu Y., Chen Z., Shen J. (2013). Occurrence and removal characteristics of phthalate esters from typical water sources in northeast china.. J. Anal. Methods Chem..

[3] Staples C.A., Parkerton T.F., Peterson D.R. (2000). A risk assessment of selected phthalate esters in North American and Western European surface waters.. Chemosphere.

[4] Benjamin S., Masai E., Kamimura N., Takahashi K., Anderson R.C., Faisal P.A. (2017). Phthalates impact human health: Epidemiological evidences and plausible mechanism of action.. J. Hazard. Mater..

[5] Yang T., Ren L., Jia Y., Fan S., Wang J., Wang J., Nahurira R., Wang H., Yan Y. (2018). Biodegradation of di-(2-ethylhexyl) phthalate by Rhodococcus ruber YC-YT1 in contaminated water and soil.. Int. J. Environ. Res. Public Health.

[6] Gadupudi C.K., Rice L., Xiao L., Kantamaneni K. (2021). Endocrine disrupting compounds removal methods from wastewater in the United Kingdom: a review.. Sci.

[7] Roccuzzo S., Beckerman A.P., Trögl J. (2021). New perspectives on the bioremediation of endocrine disrupting compounds from wastewater using algae-, bacteria- and fungi-based technologies.. Int. J. Environ. Sci. Technol..

[8] Liang D.W., Zhang T., Fang H.H.P., He J. (2008). Phthalates biodegradation in the environment.. Appl. Microbiol. Biotechnol..

[9] Cheng J., Liu Y., Wan Q., Yuan L., Yu X. (2018). Degradation of dibutyl phthalate in two contrasting agricultural soils and its long-term effects on soil microbial community.. Sci. Total Environ..

[10] Feng N.X., Feng Y.X., Liang Q.F., Chen X., Xiang L., Zhao H.M., Liu B.L., Cao G., Li Y.W., Li H., Cai Q.Y., Mo C.H., Wong M.H. (2021). Complete biodegradation of di-n-butyl phthalate (DBP) by a novel Pseudomonas sp. YJB6.. Sci. Total Environ..

[11] Yastrebova O.V., Pyankova A.A., Plotnikova E.G. (2019). Phthalate-degrading bacteria isolated from an industrial mining area and the processing of potassium and magnesium salts.. Appl. Biochem. Microbiol..

[12] Patil N.K., Karegoudar T.B. (2005). Parametric studies on batch degradation of a plasticizer di-n-butylphthalate by immobilized Bacillus sp.. World J. Microbiol. Biotechnol..

[13] Fan S., Li C., Guo J., Johansen A., Liu Y., Feng Y., Xue J., Li Z. (2023). Biodegradation of phthalic acid esters (PAEs) by Bacillus sp. LUNF1 and characterization of a novel hydrolase capable of catalyzing PAEs.. Environ. Technol. Innov..

[14] Xu Y., Liu X., Zhao J., Huang H., Wu M., Li X., Li W., Sun X., Sun B. (2021). An efficient phthalate ester-degrading Bacillus subtilis: Degradation kinetics, metabolic pathway, and catalytic mechanism of the key enzyme.. Environ. Pollut..

[15] Shariati S., Ebenau-Jehle C., Pourbabaee A.A., Alikhani H.A., Rodriguez-Franco M., Agne M., Jacoby M., Geiger R., Shariati F., Boll M. (2022). Degradation of dibutyl phthalate by Paenarthrobacter sp. Shss isolated from Saravan landfill, Hyrcanian Forests, Iran.. Biodegradation.

[16] Li C., Liu C., Li R., Liu Y., Xie J., Li B. (2022). Biodegradation of dibutyl phthalate by the New Strain Acinetobacter baumannii DP-2.. Toxics.

[17] Jin D., Kong X., Liu H., Wang X., Deng Y., Jia M., Yu X. (2016). Characterization and cenomic analysis of a highly efficient dibutyl phthalate-degrading bacterium Gordonia sp. strain QH-12.. Int. J. Mol. Sci..

[18] Liu T., Li J., Qiu L., Zhang F., Linhardt R.J., Zhong W. (2020). Combined genomic and transcriptomic analysis of the dibutyl phthalate metabolic pathway in Arthrobacter sp. ZJUTW.. Biotechnol. Bioeng..

[19] Nandi M., Paul T., Kanaujiya D.K., Baskaran D., Pakshirajan K., Pugazhenthi G. (2021). Biodegradation of benzyl butyl phthalate and dibutyl phthalate by Arthrobacter sp. *via* micellar solubilization in a surfactant-aided system.. Water Sci. Technol. Water Supply.

[20] Wang Y., Miao B., Hou D., Wu X., Peng B. (2012). Biodegradation of di-n-butyl phthalate and expression of the 3,4-phthalate dioxygenase gene in Arthrobacter sp. ZH2 strain.. Process Biochem..

[21] Wen Z.D., Gao D.W., Wu W.M. (2014). Biodegradation and kinetic analysis of phthalates by an Arthrobacter strain isolated from constructed wetland soil.. Appl. Microbiol. Biotechnol..

[22] Hu T., Yang C., Hou Z., Liu T., Mei X., Zheng L., Zhong W. (2022). Phthalate esters metabolic strain Gordonia sp. GZ-YC7, a potential soil degrader for high concentration di-(2-ethylhexyl) phthalate.. Microorganisms.

[23] Ren L., Lin Z., Liu H., Hu H. (2018). Bacteria-mediated phthalic acid esters degradation and related molecular mechanisms.. Appl. Microbiol. Biotechnol..

[24] Eaton R.W. (2001). Plasmid-encoded phthalate catabolic pathway in Arthrobacter keyseri 12B.. J. Bacteriol..

[25] Stanislauskienė R., Rudenkov M., Karvelis L., Gasparavičiūtė R., Meškienė R., Časaitė V., Meškys R. (2011). Analysis of phthalate degradation operon from Arthrobacter sp. 68b.. Biologija (Vilnius).

[26] Plotnikova E.G., Altyntseva O.V., Kosheleva I.A., Puntus I.F., Filonov A.E., Gavrish E.Y., Demakov V.A., Boronin A.M. (2001). Bacterial degraders of polycyclic aromatic hydrocarbons isolated from salt-contaminated soils and bottom sediments in salt mining areas.. Microbiology.

[27] Plotnikova E.G., Yastrebova O.V., Anan’ina L.N., Dorofeeva L.V., Lysanskaya V.Y., Demakov V.A. (2011). Halotolerant bacteria of the genus Arthrobacter degrading polycyclic aromatic hydrocarbons.. Russ. J. Ecol..

[28] Yastrebova O.V., Korsakova E.S., Plotnikova E.G. (2018). Characteristics of bacteria of Micrococcaceae family, isolated from different biotopes of salt mining area (Perm region).. Izvestia of RAS SamSC(Russia).

[29] Raymond R.L. (1961). Microbial oxidation of n-paraffinic hydrocarbons.. Dev. Ind. Microbiol..

[30] Yastrebova O.V., Malysheva A.A., Plotnikova E.G. (2022). Halotolerant terephthalic acid-degrading bacteria of the genus Glutamicibacter.. Appl. Biochem. Microbiol..

[31] Gerhardt P., Murray R.G.E., Costilow R.N., Nester E.W., Wood W.A., Krieg N.R., Phillips G (1981). Manual of methods for general bacteriology.. Amer. Soci. microbiol..

[32] Wilson K. (2001). Preparation of genomic DNA from bacteria..

[33] Andrews S. (2010). FastQC: A quality control tool for high throughput sequence data.. https://www.bioinformatics.babraham.ac.uk/projects/fastqc/.

[34] Bolger A.M., Lohse M., Usadel B. (2014). Trimmomatic: A flexible trimmer for Illumina sequence data.. Bioinformatics.

[35] Bankevich A., Nurk S., Antipov D., Gurevich A.A., Dvorkin M., Kulikov A.S., Lesin V.M., Nikolenko S.I., Pham S., Prjibelski A.D., Pyshkin A.V., Sirotkin A.V., Vyahhi N., Tesler G., Alekseyev M.A., Pevzner P.A. (2012). SPAdes: A new genome assembly algorithm and its applications to single-cell sequencing.. J. Comput. Biol..

[36] Mavromatis K., Ivanova N.N., Chen I.M.A., Szeto E., Markowitz V.M., Kyrpides N.C. (2009). The DOE-JGI standard operating procedure for the annotations of microbial genomes.. Stand. Genomic Sci..

[37] Chen F., Chen Y., Chen C., Feng L., Dong Y., Chen J., Lan J., Hou H. (2021). High-efficiency degradation of phthalic acid esters (PAEs) by Pseudarthrobacter defluvii E5: Performance, degradative pathway, and key genes.. Sci. Total Environ..

[38] Kumar V., Sharma N., Maitra S.S. (2017). Comparative study on the degradation of dibutyl phthalate by two newly isolated Pseudomonas sp. V21b and Comamonas sp. 51F.. Biotechnol. Rep..

[39] Vandera E., Samiotaki M., Parapouli M., Panayotou G., Koukkou A.I. (2015). Comparative proteomic analysis of Arthrobacter phenanthrenivorans Sphe3 on phenanthrene, phthalate and glucose.. J. Proteomics.

