# Supplementary material for: Characterization and Genomic Analysis of Arthrobacter sp. SF27: A Promising Dibutyl Phthalate-degrading Strain
Source: Curr Genomics. 2025 Mar 14;26(5):359–67. doi: 10.2174/0113892029343036250210044540 (PMC12728591; doi:10.2174/0113892029343036250210044540)
Supplement: Supplementary file 1 [file CG-26-5-359_SD1.pdf]

Supplementary Material

Characterization and Genomic Analysis of *Arthrobacter* sp. SF27: A Promising Dibutyl Phthalate-degrading Strain

Ekaterina Korsakova[1,*], Yulia Nechaeva[1], Elena Plotnikova[1] and Olga Yastrebova[1,*]

[1]*Laboratory of Microbiology of Technogenic Ecosystems, Institute of Ecology and Genetics of Microorganism UB RAS, Perm, Russia*

Table 1S. Growth of *Arthrobacter* SF27 strain on organic substrates (1 g/l).

Substrate	DBP	DMP	DEP	*ortho*-Phthalic acid	Protocatechuic acid
Maximum OD_{600}	1.15	-	0.6	1.2	0.9

Table 2S. Comparison of transcribed gene sequences of the strain *Arthrobacter* sp. SF27 involved in the degradation of phthalic acid with homologous sequence from the GenBank database.

Gene	Protein	Lenght AAS	Homologous proteins, GenBank ID, similarity (%)
phtAa	phthalate 3,4-dioxygenase, α-subunit	486	phenylpropionate dioxygenase, α-subunit [*A. crystallopoietes* DSM 20117 ^{T}], SDR29222, 97.00%; phthalate 3,4-dioxygenase, α-subunit [*A. keyseri* 12B], AAK16534, 94.00%; aromatic-ring-hydroxylating dioxygenases, α-subunit [*Pseudarthrobacter phenanthrenivorans* Sphe3], RKO19621, 94.00%
phtAb	phthalate 3,4-dioxygenase, β-subunit	201	3-phenylpropionate/cinnamic acid dioxygenase, β-субъединица [*A. crystallopoietes* DSM 20117^{T}], SDR29231, 97.00%; 3-phenylpropionate/cinnamic acid dioxygenase, β-субъединица [*P. phenanthrenivorans* Sphe3], WP_120693527, 92.00%
phtAc	phthalate 3,4-dioxygenase, ferredoxin subunit	64	phthalate 3,4-dioxygenase, ferredoxin subunit [*A. keyseri* 12B], AAK16536, 100.00%; 3-phenylpropionate/trans-cinnamate dioxygenase ferredoxin subunit [*A. crystallopoietes* DSM 20117^{T}], SDR29255, 89.00%
phtAd	phthalate 3,4-dioxygenase, ferredoxin reductase subunit	410	phthalate 3,4-dioxygenase, reductase subunit [*A. keyseri* 12B], AAK16537, 93.00%; phthalate 3,4-dioxygenase, ferredoxin reductase subunit [*P. phenanthrenivorans* Sphe3], ADX75141, 92.00%
phtB	phthalate 3,4-*cis*-dihydrodiol dehydrogenase	362	phthalate 3,4-*cis*-dihydrodiol dehydrogenase [*A. keyseri* 12B], AAK16533, 97.00%; aldo/keto reductase [*P. phenanthrenivorans* Sphe3], WP_049786163, 96.00%
phtC	3,4-dihydroxyphthalate decarboxylase	191	3,4-dihydroxyphthalate 2-decarboxylase [*A. keyseri* 12B], AAK16538, 94.00%; 3,4-dihydroxyphthalate decarboxylase [*P. phenanthrenivorans* Sphe3], ADX75142, 94.00%
phtU	hypothetical protein	112	hypothetical protein [*A. keyseri* 12B], WP_169094770, 91.00%; hypothetical protein [*P. phenanthrenivorans* Sphe3], ADX75139, 90.00%;
phtR	IclR family transcriptional regulator	283	IclR family transcriptional regulator [*P. phenanthrenivorans* Sphe3], WP_041653696, 90.00%; putative *pht* operon regulator [*A. keyseri* 12B], AAK16539, 86.00%

Table 3S. Comparison of transcribed gene sequences of the strain *Arthrobacter* sp. SF27 involved in the degradation of aromatic compounds with homologous sequences from the GenBank database.

Gene	GenBank ID	Length (bp)	Protein	Homologous proteins, GenBank ID, similarity (%)
**Protocatechuate branch of *β*-ketoadipate pathway**				
pcaG	WP_169993267	573	protocatechuate 3,4-dioxygenase alpha subunit	protocatechuate 3,4-dioxygenase subunit alpha [*Arthrobacter crystallopoietes* DSM 20117^T], WP_074701271, 96.00%
pcaH	WP_169993268	846	protocatechuate 3,4-dioxygenase beta subunit	protocatechuate 3,4-dioxygenase subunit beta [*A. crystallopoietes* DSM 20117^T], WP_139005728, 98.90%
pcaB	WP_169993265	1425	3-carboxy-*cis*, *cis*-muconate cycloisomerase	lyase family protein [*Arthrobacter* sp. VKM Ac-2550] WP_264668651, 95.78%
pcaC	NMR29463	426	4-carboxymuconolactone decarboxylase	4-carboxymuconolactone decarboxylase [*A. crystallopoietes* DSM 20117^T], WP_074701274, 96.45%
pcaD	WP_169993264	795	3-oxoadipate enol-lactonase	alpha/beta fold hydrolase [*A. crystallopoietes* DSM 20117^T], WP_074701273, 96.97%
pcaI	WP_169993259	699	3-oxoadipate CoA-transferase alpha subunit	3-oxoacid CoA-transferase subunit A [*Arthrobacter* sp. VKM Ac-2550], WP_264668647, 99.57%
pcaJ	NMR29460	642	3-oxoadipate CoA-transferase beta subunit	3-oxoacid CoA-transferase subunit B [*A. crystallopoietes* DSM 20117^T], WP_208574859, 98.59%
**Benzoate degradation**				
benA	WP_169999089	1179	benzoate/toluate 1,2-dioxygenase alpha subunit	benzoate 1,2-dioxygenase large subunit [*A. crystallopoietes* DSM 20117^T], WP_074700825, 98.22%; benzoate 1,2-dioxygenase large subunit [*Arthrobacter* sp. VKM Ac-2550]; WP_264671044, 97.46%
benB	WP_169996777	534	benzoate/toluate 1,2-dioxygenase beta subunit	benzoate 1,2-dioxygenase small subunit [*A. crystallopoietes* DSM 20117^T], WP_139007136, 98.31%; benzoate 1,2-dioxygenase small subunit [*Arthrobacter* sp. VKM Ac-2550], WP_264671043, 97.18%
	WP_169997261	519	benzoate/toluate 1,2-dioxygenase beta subunit	benzoate 1,2-dioxygenase small subunit [*A. crystallopoietes* DSM 20117^T], WP_074700826, 97.67%
benC	WP_169996779	1035	benzoate/toluate 1,2-dioxygenase reductase subunit	benzoate 1,2-dioxygenase electron transfer component BenC [*A. crystallopoietes* DSM 20117^T], WP_139007135, 99.71%; benzoate 1,2-dioxygenase electron transfer component BenC [*Arthrobacter* sp. VKM Ac-2550], WP_264671042, 99.42%
	WP_169997263	1092		benzoate 1,2-dioxygenase electron transfer component BenC [*A. crystallopoietes* DSM 20117^T], WP_139004252, 98.35% benzoate 1,2-dioxygenase electron transfer component BenC [*Arthrobacter* sp. VKM Ac-2550], WP_264671497, 98.07%
benD	WP_169996781	825	1,6-dihydroxycyclohexa-2,4-diene-1-carboxylate dehydrogenase	1,6-dihydroxycyclohexa-2,4-diene-1-carboxylate dehydrogenase [*A. crystallopoietes* DSM 20117^T], WP_074700715, 96.35%
benE	WP_240974387	1146	benzoate membrane transport protein	benzoate membrane transport protein [*Arthrobacter* sp. VKM Ac-2550] SMP98222, 99.25%

	WP_169997602	1224		benzoate membrane transport protein [*Arthrobacter* sp. VKM Ac-2550] SMP99117, 98.77%
	WP_169996006	1254		benzoate/H(+) symporter BenE family transporter [*Arthrobacter* sp. VKM Ac-2550] WP_264670889, 98.08%
benK	WP_169997265	1398	benzoate transport protein	aromatic acid/H+ symport family MFS transporter [*Arthrobacter* sp. VKM Ac-2550] WP_264671498, 97.85%
**Catechol branch of *β*-ketoadipate pathway**				
catA	WP_169991363	846	catechol 1,2-dioxygenase	catechol 1,2-dioxygenase [*Arthrobacter* sp. VKM Ac-2550], WP_264670356, 99.29%; catechol 1,2-dioxygenase [*A. crystallopoietes* DSM 20117^T], WP_074699384, 96.80%
	WP_169998093	849		catechol 1,2-dioxygenase [*Arthrobacter* sp. VKM Ac-2550], WP_264670356, 99.29%; catechol 1,2-dioxygenase [*A. crystallopoietes* DSM 20117^T], WP_074699384, 98.58%
catE	WP_169996191	885	catechol 2,3-dioxygenase	3,4-dihydroxyphenylacetate 2,3-dioxygenase [*A. crystallopoietes* DSM 20117^T], WP_208574682, 98.00% catechol 1,2-dioxygenase [*Pseudarthrobacter phenanthrenivorans* J015], RKO19277, 100.00% VOC family protein [*Arthrobacter* sp. VKM Ac-2550], WP_264668375, 97.00%
**Aromatic compounds degradation (phenanthrene, napthalene)**				
nidA	WP_169998856	1449	nitrite reductase/ring-hydroxylating ferredoxin subunit (PAH dioxygenase large subunit)	Rieske 2Fe-2S domain-containing protein [*Pseudarthrobacter phenanthrenivorans* Sphe3] WP_013602971, 99.17%
phdJ	WP_240974903	1110	dihydrodipicolinate synthase/N-acetylneuraminate lyase (4-(2-carboxyphenyl)-2-oxobut-3-enoate aldolase)	dihydrodipicolinate synthase family protein [*Pseudarthrobacter phenanthrenivorans* Sphe3] WP_013602975, 99.40%
phdK	WP_205834947	1455	2-formylbenzoate dehydrogenase	aldehyde dehydrogenase family protein [*Pseudarthrobacter phenanthrenivorans* Sphe3] WP_013602981, 99.38%
phdI	WP_169998706	1164	1-hydroxy-2-naphthoate 1,2-dioxygenasee (gentisate 1,2-dioxygenase)	1-hydroxy-2-naphthoate dioxygenase [*Pseudarthrobacter phenanthrenivorans* Sphe3] WP_013602983, 99.74%
phdI	WP_169998731	1164	1-hydroxy-2-naphthoate 1,2-dioxygenasee (gentisate 1,2-dioxygenase)	1-hydroxy-2-naphthoate dioxygenase [*Pseudarthrobacter phenanthrenivorans* Sphe3] WP_013601271, 91.73%

Table 4S. Comparison of translated amino acid sequences of alpha/beta hydrolase in the strain *Arthrobacter* sp. SF27 with homologous amino acid sequences of genes from the GenBank database.

Protein	GenBank ID	Length (bp)	Homologous proteins, GenBank ID, similarity (%)
alpha/beta hydrolase	NMR28165	450	alpha/beta hydrolase [*A crystallopoietes* DSM 20117^T], WP_139005298, 81.33% alpha/beta hydrolase [*Arthrobacter* sp. Bi26], WP_229993686, 78.67%

alpha/beta hydrolase	NMR28342	259	alpha/beta hydrolase [*Arthrobacter* sp. VKM Ac-2550], WP_264669012, 97.68%<br>alpha/beta hydrolase [*A. crystallopoietes* DSM 20117^T], WP_074699262, 96.53%
alpha/beta hydrolase	NMR28537	279	alpha/beta hydrolase [*A. crystallopoietes* DSM 20117^T], WP_139005298, 98.57%<br>alpha/beta hydrolase [*Arthrobacter* sp. VKM Ac-2550], WP_264670680, 97.84%
alpha/beta hydrolase	NMR28183	285	pimeloyl-ACP methyl ester carboxylesterase [*Arthrobacter* sp. VKM Ac-2550], MCW2132372, 93.00%<br>alpha/beta hydrolase [*Arthrobacter* sp. VKM Ac-2550], WP_264668882, 93.33%<br>alpha/beta hydrolase [*A. crystallopoietes* DSM 20117^T], WP_074699150, 91.13%
alpha/beta hydrolase	NMR28662	382	lysophospholipase [*Arthrobacter* sp. VKM Ac-2550], WP_264671393, 95.80%<br>alpha/beta fold hydrolase [*A. crystallopoietes* DSM 20117^T], WP_139186743, 91.05%
alpha/beta hydrolase	NMR28874	253	alpha/beta hydrolase [*Arthrobacter* sp. VKM Ac-2550], WP_264667762, 95.63%<br>alpha/beta hydrolase [*A. crystallopoietes* DSM 20117^T], WP_208575329, 92.49%
alpha/beta hydrolase	NMR28880	217	dienelactone hydrolase [*Arthrobacter* sp. VKM Ac-2550], WP_264667757, 92.00%<br>dienelactone hydrolase family protein [*A. crystallopoietes* DSM 20117^T], WP_074700243, 88.89%
alpha/beta hydrolase	NMR29000	336	alpha/beta hydrolase [*Arthrobacter* sp. VKM Ac-2550], WP_264667647, 98.51%<br>alpha/beta hydrolase [*A. crystallopoietes* DSM 20117^T], WP_074700136, 96.13%
alpha/beta hydrolase	NMR29240	232	alpha/beta hydrolase [*Arthrobacter* sp. VKM Ac-2550], WP_264669562, 96.98%<br>alpha/beta hydrolase [*A. crystallopoietes* DSM 20117^T], WP_139004133, 91.38%
alpha/beta hydrolase	NMR29540	240	alpha/beta hydrolase [*Arthrobacter* sp. VKM Ac-2550], WP_264668715, 94.58%<br>alpha/beta hydrolase [*A. crystallopoietes* DSM 20117^T], AUI49778, 93.75%
alpha/beta hydrolase	NMR29541	247	alpha/beta hydrolase [*Arthrobacter* sp. VKM Ac-2550], WP_264668716, 97.98%<br>alpha/beta hydrolase [*A. crystallopoietes* DSM 20117^T], WP_074701206, 93.12%
alpha/beta hydrolase	NMR30209	431	alpha/beta hydrolase family protein [*Arthrobacter* sp. VKM Ac-2550], MCW2133351, 99.07%<br>alpha/beta fold hydrolase [*A. crystallopoietes* DSM 20117^T], WP_139003689, 97.68%
alpha/beta hydrolase	NMR30273	441	alpha/beta hydrolase [*Arthrobacter* sp. VKM Ac-2550], WP_264669436, 95.46%<br>alpha/beta fold hydrolase [*A. crystallopoietes* DSM 20117^T], WP_139003805, 92.06%
alpha/beta hydrolase	NMR30477	260	Pimeloyl-ACP methyl ester carboxylesterase [*Arthrobacter* sp. VKM Ac-2550], MCW2131568, 98.85%<br>alpha/beta hydrolase [*A. crystallopoietes* DSM 20117^T], WP_171059408, 94.23%
alpha/beta hydrolase	NMR30501	472	alpha/beta hydrolase [*Arthrobacter* sp. VKM Ac-2550], WP_264668209, 93.43%<br>alpha/beta hydrolase [*A. crystallopoietes* DSM 20117^T], WP_074698953, 89.55%

alpha/beta hydrolase	NMR30736	243	alpha/beta hydrolase [*Arthrobacter* sp. VKM Ac-2550], WP_264670179, 95.45%<br>alpha/beta hydrolase [*A. crystallopoietes* DSM 20117^{T}], WP_074701024, 95.04%
alpha/beta hydrolase	NMR30861	350	alpha/beta hydrolase [*Arthrobacter* sp. VKM Ac-2550], WP_264670585, 97.14%<br>alpha/beta hydrolase [*A. crystallopoietes* DSM 20117^{T}], WP_139004367, 95.71%
alpha/beta hydrolase	NMR30992	236	alpha/beta hydrolase [*Arthrobacter* sp. VKM Ac-2550], WP_264668373, 97.03%<br>alpha/beta hydrolase [*A. crystallopoietes*] DSM 20117^{T}, WP_074702042, 95.74%
alpha/beta hydrolase	NMR31428	276	alpha/beta hydrolase [*A. crystallopoietes* DSM 20117^{T}], WP_208574095, 82.76 %<br>alpha/beta fold hydrolase [*A. crystallopoietes* DSM 20117^{T}], WP_240793131, 86.55%
alpha/beta hydrolase	NMR31676	355	alpha/beta hydrolase [*Arthrobacter* sp. VKM Ac-2550], WP_264670275, 99.15%<br>alpha/beta hydrolase [*A. crystallopoietes* DSM 20117^{T}], WP_074702328, 96.62%
alpha/beta hydrolase	NMR31703	257	alpha/beta hydrolase [*Arthrobacter* sp. VKM Ac-2550], WP_264668546, 96.50%<br>alpha/beta hydrolase [*A. crystallopoietes* DSM 20117^{T}], WP_139003471, 94.55%
alpha/beta hydrolase	NMR32001	268	alpha/beta fold hydrolase [*Arthrobacter* sp. VKM Ac-2550], WP_264671329, 99.63%<br>alpha/beta fold hydrolase [*A. crystallopoietes* DSM 20117^{T}], WP_208574678, 97.39%

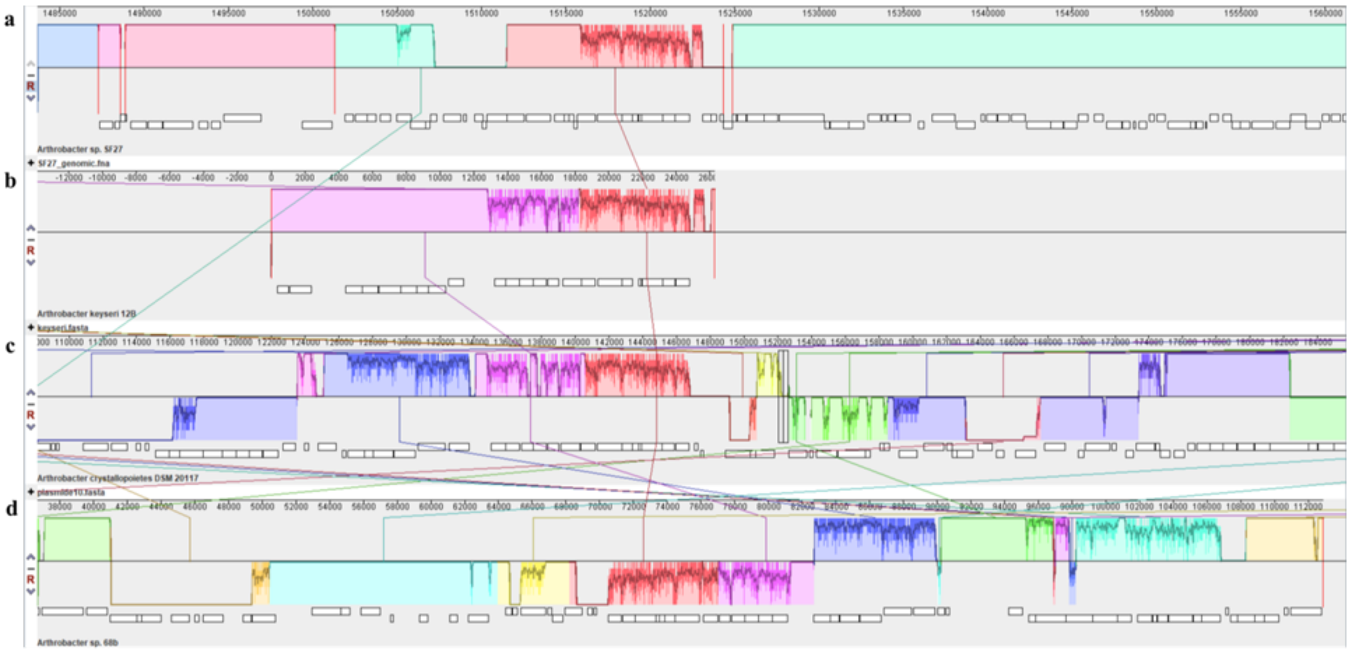

Fig. (1S). Mauve visualization of locally collinear blocks identified between chromosomes of *Arthrobacter* sp. SF27 (**a**), *Arthrobacter crystallopoietes* DSM 20117^{T} (**b**), plasmids pRE1 from *Arthrobacter keyseri* 12B (**c**), p2MP from *Arthrobacter* sp. 68b (**d**).
